# PBDE: Structure-Activity Studies for the Inhibition of Hepatitis C Virus NS3 Helicase

**DOI:** 10.3390/molecules19044006

**Published:** 2014-04-02

**Authors:** Kazi Abdus Salam, Atsushi Furuta, Naohiro Noda, Satoshi Tsuneda, Yuji Sekiguchi, Atsuya Yamashita, Kohji Moriishi, Masamichi Nakakoshi, Hidenori Tani, Sona Rani Roy, Junichi Tanaka, Masayoshi Tsubuki, Nobuyoshi Akimitsu

**Affiliations:** 1 Radioisotope Center, The University of Tokyo, 2-11-16 Yayoi, Bunkyo-ku, Tokyo 113-0032, Japan; E-Mail: salam_bio26@yahoo.com; 2 Department of Life Science and Medical Bioscience, Waseda University, 2-2 Wakamatsu-cho, Shinjuku-ku, Tokyo 162-8480, Japan; E-Mails: atsushi.5961@ruri.waseda.jp (A.F.); stsuneda@waseda.jp (S.T.); 3 Biomedical Research Institute, National Institute of Advanced Industrial Science and Technology (AIST), 1-1-1 Higashi, Tsukuba, Ibaraki 305-8566, Japan; E-Mails: noda-naohiro@aist.go.jp (N.N.); y.sekiguchi@aist.go.jp (Y.S.); 4 Department of Microbiology, Graduate School of Medicine and Engineering, University of Yamanashi, 1110 Shimokato, Chuo-shi, Yamanashi 409-3898, Japan; E-Mails: atsuyay@yamanashi.ac.jp (A.Y.); kmoriishi@yamanashi.ac.jp (K.M.); 5 Faculty of Pharmaceutical Sciences, Toho University, 2-2-1 Miyama, Funabashi, Chiba 274-8510, Japan; E-Mail: nakakoshi@phar.toho-u.ac.jp; 6 Research Institute for Environmental Management Technology, National Institute of Advanced Industrial Science and Technology (AIST), 16-1, Onogawa, Tsukuba, Ibaraki 305-8569, Japan; E-Mail: h.tani@aist.go.jp; 7 Department of Chemistry, Biology and Marine Science, University of the Ryukyus, Nishihara, Okinawa 903-0213, Japan; E-Mails: sonarroy@gmail.com (S.R.R.); jtanaka@sci.u-ryukyu.ac.jp (J.T.); 8 Institute of Medical Chemistry, Hoshi University, Ebara 2-4-41, Shinagawa-ku, Tokyo 142-8501, Japan

**Keywords:** hepatitis C virus, NS3 RNA helicase, marine sponge, polybrominated diphenyl ether

## Abstract

The helicase portion of the hepatitis C virus nonstructural protein 3 (NS3) is considered one of the most validated targets for developing direct acting antiviral agents. We isolated polybrominated diphenyl ether (PBDE) **1** from a marine sponge as an NS3 helicase inhibitor. In this study, we evaluated the inhibitory effects of PBDE (**1**) on the essential activities of NS3 protein such as RNA helicase, ATPase, and RNA binding activities. The structure-activity relationship analysis of PBDE (**1**) against the HCV ATPase revealed that the biphenyl ring, bromine, and phenolic hydroxyl group on the benzene backbone might be a basic scaffold for the inhibitory potency.

## 1. Introduction

Hepatitis C virus (HCV) is one of the major causative agents for hepatitis C, which has caused an epidemic of liver fibrosis, cirrhosis, and hepatocellular carcinoma [1]. HCV infects more than 150 million people worldwide, and over 350,000 people die from HCV-related liver diseases each year [2]. The virus is undetectable for long periods of time, even decades, and replicates slowly without major complications. Therefore, most infected people are unaware they carry the virus. HCV is only transmitted via blood and blood products, while sexual and mother-to-child transmission is much less likely than for HIV infection [2].

The recently approved new treatment regimen for HCV infection is the combination of pegylated interferon and ribavirin with either telaprevir or boceprevir for genotype 1 infected patients. However, the emergence of viral resistance to the drugs as well as side effects, such as anemia, neutropenia, dysgeusia, rash, and anorectal discomfort, are the main concerns [3,4,5,6]. Despite intensive studies for the development of new antiviral drugs, HCV is still a major threat to human health. Therefore, there is an urgent need to develop new antiviral drugs with fewer side effects and the highest antiviral efficacy.

HCV is a single-stranded, positive-sense RNA virus in the *Flaviviridae* family [1,7]. Seven genotypes and more than 50 subtypes of HCV have been described [8]. The viral genome is 9.6 kb in length and contains one main open reading frame encoding an approximately 3,000 amino acid single polyprotein, flanked by a 5'-non-translated region (NTR) and a 3'-NTR. Once translation initiated by an internal ribosome entry site present at the 5'-NTR, host and viral proteases cleave the product into 10 individual viral mature proteins [9]. The structural proteins (envelope glycoproteins; E1 and E2) are responsible for receptor binding, thereby facilitating viral entry into the hepatocyte. The core protein (C) forms the viral nucleocapsid [10]. The nonstructural proteins p7, NS2, NS3, NS4A, NS4B, NS5A, and NS5B are involved in viral replication and packaging of the HCV genome. NS3 is a multifunctional protein that plays an important role in the viral life cycle. It has a serine protease (NS3/4A) activity at the N-terminal to cleave all downstream junctions, and helicase activity at the C-terminal to separate double-stranded RNA in a reaction fueled by ATP hydrolysis during replication of viral genomic RNA [11,12]. Although the precise role of the helicase activity in the viral life cycle is not well understood, a fully functional helicase is essential for HCV RNA replication. The helicase portion of NS3 is thus a valid target for the development of direct acting antiviral therapy.

The development of antiviral agents for the treatment of HCV infection has been focused on small molecule inhibitors of HCV infection that can act directly on viral targets or other host target proteins critical to HCV replication. The first two approved direct acting antiviral agents, telaprevir and boceprevir, are inhibitors of the NS3/4A protease activity [13]. However, very few compounds that inhibit the NS3 helicase function have been reported, and to the best of our knowledge, no helicase inhibitors have entered clinical trials. Thus there is still a great need in HCV research to develop novel NS3 helicase inhibitors.

The aim of this project was to identify a possible NS3 helicase inhibitor from marine natural products. In this study, we successfully obtained from marine sponge and identified hydroxylated polybrominated diphenyl ether OH-PBDE-47 (**1**), as a helicase inhibitor through a high-throughput screening method based on fluorescence resonance energy transfer (FRET). We also evaluated several commercially available compounds that are structurally related to PBDE (**1**) for the study of structure-activity relationships.

PBDEs have been found to exhibit antibacterial, antifungal, and antimicroalgal activities [14,15,16,17,18,19]. They inhibit a wide range of enzymes that are relevant to anticancer drug discovery such as inosine monophosphate dehydrogenase, guanosine monophosphate synthetase, and 15-lipoxygenase [20]. PBDEs have also been shown to exhibit inhibitory activities against the assembly of microtubule protein, the maturation of starfish oocytes [21] and Tie2 kinase [22]. In this research, we found a novel activity of PBDE in the specific inhibition of HCV NS3 helicase activity.

## 2. Results and Discussion

**Figure 1:**
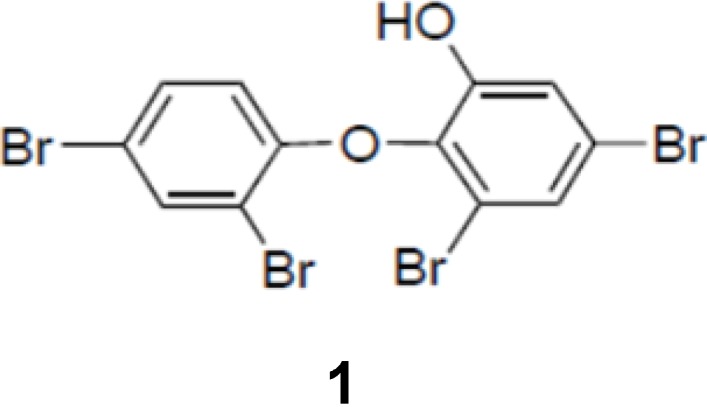
Chemical structure of PBDE (**1**).

To screen potential NS3 helicase inhibitors from extracts of marine organisms, we used a high-throughput fluorescence helicase assay based on FRET [23]. Out of 41 extracts isolated (Table 1), PBDE (**1**) (Figure 1) exhibited the strongest inhibition (37%) of NS3 helicase activity.

**Table 1:** Inhibitory effects of extracts from marine organisms on hepatitis C virus (HCV) nonstructural protein 3 (NS3) helicase activity.

No.	Sample ID	FRET (%) ^a^	Possibly Contained Molecule	Species	Location
1	PM-35-1	85	misakinolide	sponge (*Theonella* sp.)	Tokashiki Island, Okinawa
2	PM-35-2	94		sponge (*Theonella* sp.)	Tokashiki Island, Okinawa
3	PM-36-1	79		gorgonian (*Euplexaura* sp.)	Tokashiki Island, Okinawa
4	PM-36-2	114		gorgonian (*Euplexaura* sp.)	Tokashiki Island, Okinawa
5	PM-37-1	93	briarane diterpenes	gorgonian (*Junceella fragilis*)	Tokashiki Island, Okinawa
6	PM-37-2	115		gorgonian (*Junceella fragilis*)	Tokashiki Island, Okinawa
7	PM-38-1	92	hippuristanol	gorgonian (*Isis hippuris*)	Tokashiki Island, Okinawa
8	PM-38-2	112		gorgonian (*Isis hippuris*)	Tokashiki Island, Okinawa
9	PM-39-2	94		sponge (*Petrosia* sp.)	Tokashiki Island, Okinawa
10	SR-1-1	37	PBDE	sponge (*Dysidea granulosa*)	Yonaguni Island, Okinawa
11	SR-2-2	92		sponge (*Jaspis* sp.)	Yonaguni Island, Okinawa
12	SR-3-1	75	petrosynol/petrosynone	sponge (*Petrosia* sp.)	Yonaguni Island, Okinawa
13	SR-4-1	68	strongylophorines	sponge (*Strongylophora* sp.)	Yonaguni Island, Okinawa
14	SR-4-2	86		sponge (*Strongylophora* sp.)	Yonaguni Island, Okinawa
15	SR-6-1	98	sesquiterpenes	soft coral (*Clavularia* sp.)	Yonaguni Island, Okinawa
16	SR-8-1	98		soft coral (*Parerythropodium* sp.)	Yonaguni Island, Okinawa
17	SR-8-2	84		Yonaguni Island, Okinawa	Yonaguni Island, Okinawa
18	SR-10-1	112	polyketide peroxides	sponge (*Plakortis* sp.)	Yonaguni Island, Okinawa
19	SR-11-1	65		sponge (unidentified)	Yonaguni Island, Okinawa
20	SR-12-2	135		sponge (unidentified)	Yonaguni Island, Okinawa
21	SR-13-2	109		sponge (*Pseudoceratina purpurea*)	Yonaguni Island, Okinawa
22	SR-14-1	64	swinholide	sponge (*Theonella swinhoei*)	Yonaguni Island, Okinawa
23	SR-15-1	61		sponge (unidentified)	Yonaguni Island, Okinawa
24	SR-16-1	92		sponge (unidentified)	Yonaguni Island, Okinawa
25	SR-17-2	87		sponge (unidentified)	Yonaguni Island, Okinawa
26	SR-19-2	131		sponge (*Hyrtios* sp.)	Yonaguni Island, Okinawa
27	SR-21-1	155	xestospongin	sponge (*Xestospongia* sp.)	Yonaguni Island, Okinawa
28	SR-21-2	156		sponge (*Xestospongia* sp.)	Yonaguni Island, Okinawa
29	SR-23-1	73	avarol	sponge (*Dysidea arenaria*)	Yonaguni Island, Okinawa
30	SR-23-2	82		sponge (*Dysidea arenaria*)	Yonaguni Island, Okinawa
31	SR-24-1	123	isocyanosesquiterpenes	sponge (*Theonella* sp.)	Yonaguni Island, Okinawa
32	SR-26-2	102		sponge (unidentified)	Yonaguni Island, Okinawa
33	SR-27-1	188		sponge (*Leucetta* sp.)	Yonaguni Island, Okinawa
34	SR-27-2	171		sponge (*Leucetta* sp.)	Yonaguni Island, Okinawa
35	SR-28-1	107		sponge (unidentified)	Yonaguni Island, Okinawa
36	SR-29-2	145		sponge (*Aaptos* sp.)	Yonaguni Island, Okinawa
37	SR-30-2	215	agelasine	sponge (*Agelas* sp.)	Yonaguni Island, Okinawa
38	SR-31-1	100	hippuristanol	gorgonian	Yonaguni Island, Okinawa
				(*Isis hippuris*)	
39	SR-33-2	88		sponge (unidentified)	Yonaguni Island, Okinawa
40	SR-34-1	125		zoanthus	Yonaguni Island, Okinawa
				(*Palythoa* sp.)	
41	SR-34-2	124	palytoxin	zoanthus	Yonaguni Island, Okinawa
				(*Palythoa* sp.)	

^a^ NS3 activity in the presence of marine organisms extract is expressed as a percentage of the control in the absence of extract (100%).

**Figure 2:**
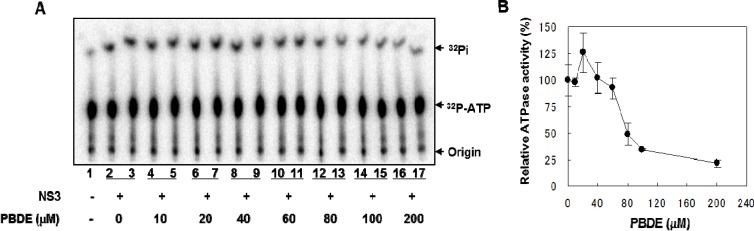
PBDE (**1**) inhibits NS3 ATPase activity. (**A**) Radioisotope labeling ATPase assay with NS3 (300 nM) and various concentrations of PBDE. Lane 1 shows the negative control reaction. Lanes 2–3 show the reaction mixture containing only NS3 and DMSO. Lanes 4–17 show hydrolytic reactions with NS3 (300 nM) in the presence of PBDE as indicated. (**B**) Graphical representation of the inhibition results. The relative ATPase activity for control reactions was considered as 100%. The average values are presented with error bars from duplicate assays.

NS3 helicase hydrolyzes ATP as an energy source to drive the unwinding of dsRNA or dsDNA. Therefore, we measured the inhibitory effects of PBDE (**1**) on the ATPase activity of the helicase portion of NS3. A radioisotope labeling ATPase assay showed that PBDE (**1**) inhibited the hydrolytic release of inorganic phosphate from ATP with an IC_50_ of 80 μM (Figure 2A, B).

**Figure 3:**
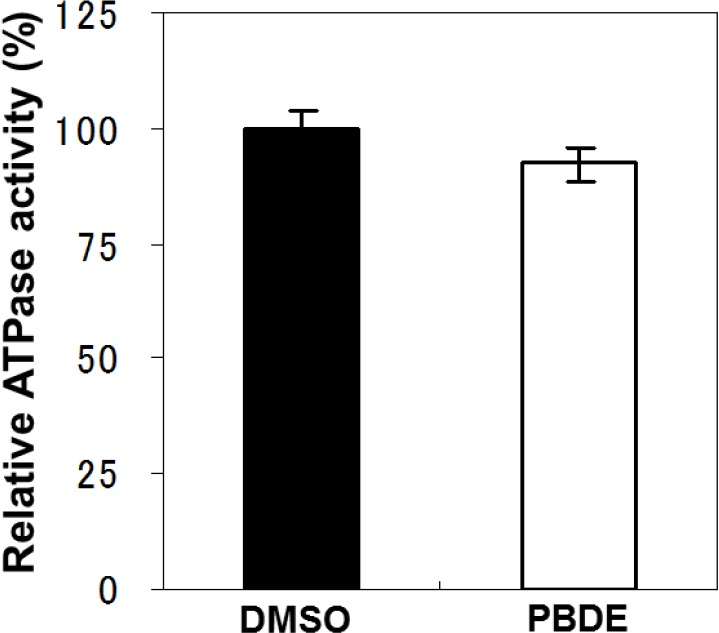
Effect of PBDE (**1**) on the ATPase activity of bacterial alkaline phosphatase. The assay was conducted in the absence (DMSO) or presence of PBDE (**1**) (at the highest concentration tested, 200 μM). The data are expressed as the mean of three replicates with error bars representing standard deviation.

To examine the specificity of PBDE (**1**) for the inhibition of ATPase activity, we evaluated the ATP hydrolytic effect on bacterial alkaline phosphatase. PBDE (**1**) exhibited no inhibition (Figure 3), indicating that the inhibitory activity of PBDE (**1**) is specific to NS3. 

**Figure 4:**
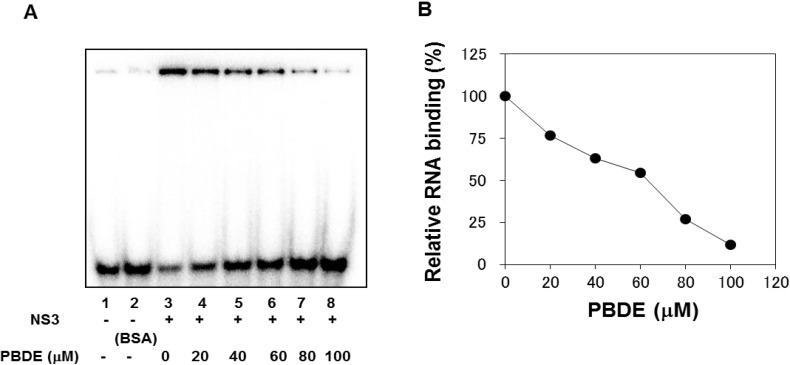
PBDE (**1**) inhibits NS3 RNA binding. (**A**) Gel mobility shift assay to characterize the inhibition of NS3 binding to [γ-^32^P] labeled ssRNA. RNA only control (lane 1), 300 nM BSA instead of NS3 control (lane 2), NS3 protein (300 nM) and DMSO control (lane 3), and NS3 protein with increasing concentrations of PBDE (**1**) (lanes 4–8). (**B**) Graphical representation of the RNA binding inhibition shown in panel A.

The binding of NS3 to ssRNA is required to initiate the unwinding activity of dsRNA during viral replication. We employed a gel mobility shift assay to characterize the inhibition of NS3 binding to RNA. PBDE (**1**) inhibited RNA binding of NS3 in a dose-dependent manner with an IC_50_ of 68 μM (Figure 4A,B). Previous reports indicate that poly(U) RNA enhances the ATPase activity of NS3 [24]. Because PBDE (**1**) inhibits the RNA binding ability of NS3, we speculated that inhibition of NS3 ATPase activity by PBDE (**1**) could be mediated through the inhibition of poly(U) RNA binding.

**Figure 5:**
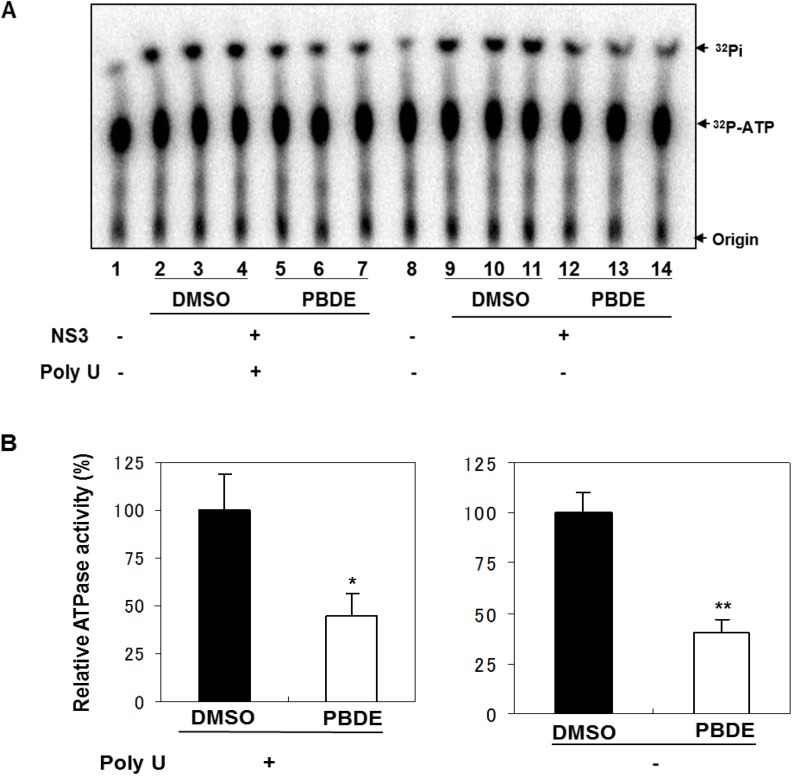
Effect of poly(U) RNA on NS3 ATPase activity. (**A**) ATPase assay with hydrolytic reaction buffer containing NS3 (600 nM), 1 mM [γ-^32^P] ATP, poly(U) RNA and PBDE (0.1 mM) as indicated. (**B**) Graphical representation of data presented in (A). The solid and white bars represent NS3 and poly(U) ATPase reactions performed with DMSO and PBDE (**1**), respectively. The assay was performed in triplicate and data are presented as mean ± standard deviation. * *p* > 0.05 and ** *p* > 0.01 from Student *t*-test.

Therefore, we next performed ATPase assays including poly(U) RNA to determine the effects of poly(U) with PBDE (**1**) near to its IC_50_ concentration. We found that PBDE (**1**) was significantly active in both the presence and absence of poly(U) (Figure 5A,B), suggesting that poly(U) has no effect on the ATPase inhibition mediated by PBDE (**1**). These results are consistent with our previous data (Figure 2B).

To clarify the structure-activity relationships of PBDE for inhibition of the ATPase activity of the NS3 protein, commercially available and natural phenol derivatives were examined (Table 2). We first investigated whether the hydroxyl group of PBDE (**1**) is required for ATPase activity. Substituting a methoxy group [25] (*i.e.*, PBDE methyl ether **2**) and a hydrogen (*i.e.*, deoxy PBDE **17**), for a phenolic hydroxyl group in PBDE led to a complete loss of the inhibitory activity. These findings indicated that the phenolic hydroxyl group has important effects on the inhibitory activity. Triclosan (**4**), which is structurally very close to PBDE, showed moderate levels of inhibition, indicating that bromine substituents on benzene rings can be replaced by chlorine substituents.

**Table 2:** Inhibition of the ATPase activity of the NS3 protein by PBDE (**1**) and its structurally related compounds.

Compound No.	Chemical Structure	NS3 ATPase Inhibition	
	(PBDE/related compounds)	IC_50_ (μM)	
1	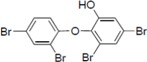	80	
2	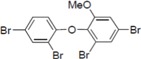	>200	
3	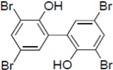	94	
4	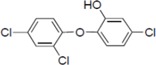	150	
5	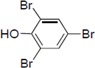	>200	
6	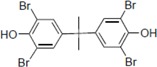	120	
7	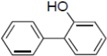	>200	
8	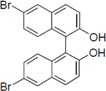	54	
9	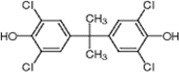	94	
10	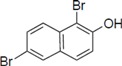	>200	
11	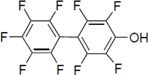	>200	
12	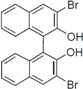	>200	
13	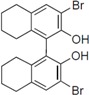	>200	
14	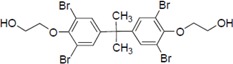	>200	
15	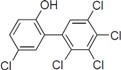	54	
16	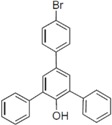	>200	
17	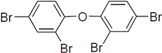	>200	

Next, we took into consideration the size of the structural motif [biphenyl (compounds **3**,**7**,**11**,**15**) compared to phenyl (compound **5**) and fused ring (compound **10**)]. Interestingly, the inhibitory activity of bromophene **3**, a biphenyl derivative possessing bromine and phenolic hydroxyl groups, remained at the same level as that of PBDE (**1**). While *o*-hydroxybiphenyl **7** showed loss of the inhibitory activity, hydroxyl-pentachlorobiphenyl **15** displayed the most potent inhibitory activity of all the analogs in this study. Notably, an additional halogen substituent on the benzene ring led to a nearly two-fold increase in activity over bromophene **3**.

Furthermore, hydroxynonafluorobiphenyl **11** was also inactive. These findings suggested that both halogen, such as bromine and chlorine, and phenolic hydroxyl groups on benzene rings would be crucial for the inhibition of the ATPase activity of the NS3 protein. Unfortunately, tribromophenol **5** and dibromonaphthalenol **10** did not exhibit the inhibitory activity, indicating that the molecular frame could affect the activity level. Tetrahalobisphenols A, (compounds **6** and **9**), showed the same level of inhibition as that of bromophene **3**. Replacement of methoxy groups in tetrabromobisphenol A (**6**) with 2-hydroxyethoxy groups, *i.e.*, the bisphenol A hydroxyethyl ether **14**, brought about loss of activity. Dibromobinaphthol **8**, a dimer of bromonaphthalenol **10**, displayed the most potent activity, whereas isomeric bromobinaphthol **12** and tetrahydrobromobinaphthol **13** showed no activity. These findings indicated that the distance between halogen and phenolic hydroxyl groups has important effects on the inhibitory activity. 4-Bromophenyl-2,6-diphenylphenol **16** did not show inhibitory activity likely because of the steric hindrance around the phenolic hydroxyl group.

**Figure 6:**
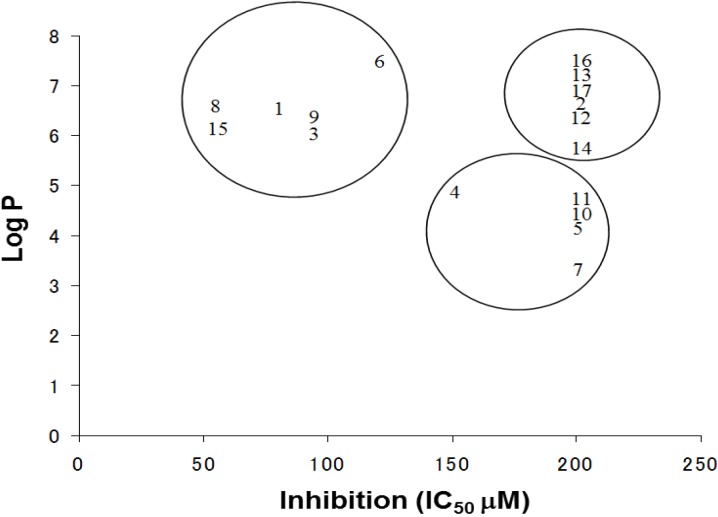
The relationships between Log P and IC_50_ values of the compounds.

The log P is a measure of the lipophilicity of an organic compound, and can be defined as the ratio of the concentration of the unionized compound at equilibrium between organic and aqueous phases. Studies have shown that many biological phenomena can be correlated with this parameter, such that structure-activity relationships may be deduced. The relationship between the IC_50_ and log P of PBDE and its structurally related compounds **1**–**17** is shown in Figure 6. Biphenyl ethers **1** and **4**, biphenyls **3** and **15**, tetrahalobisphenols A **6** and **9**, and binaphthol **8** with a log P of over approximately 5 were located at the upper left, indicating the inhibitory activity. The inhibitory potency of halogenated phenols on the ATPase might increase with growing lipophilicity. Therefore, we have identified PBDE (**1**) and related compounds, hydroxypentachlorobiphenyl and dibromobinaphthol, as potent inhibitors of the HCV ATPase. 

**Figure 7:**
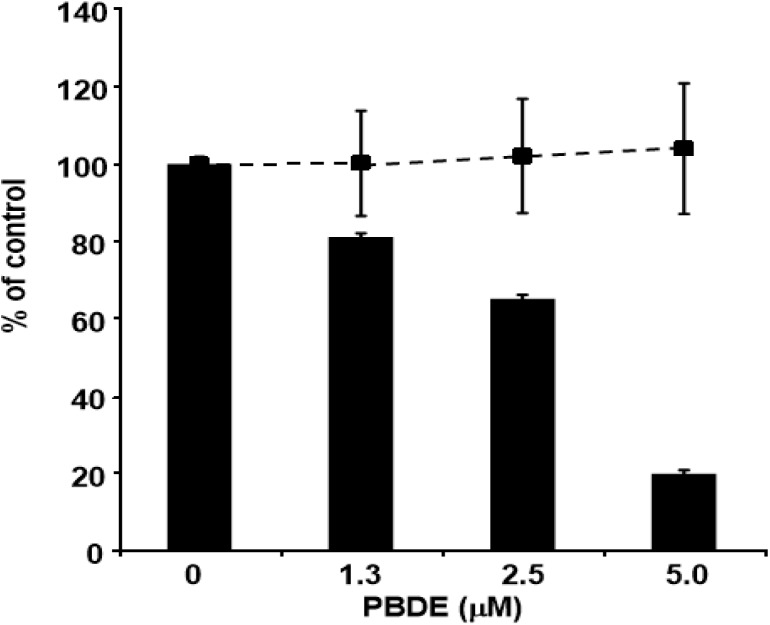
Effect of PBDE (**1**) on viral replication. The subgenomic replicon RNA of genotype 1b N strain was incubated in medium containing various concentrations of PBDE (**1**) or DMSO. Luciferase and cytotoxicity assays were carried out as described in Experimental section. Error bars indicate standard deviation. The data represent three independent experiments.

Finally, we examined the effects of PBDE (**1**) on HCV replication. As shown in Figure 7, PBDE (**1**) suppressed HCV replication in a dose-dependent manner (EC_50_ = 3.3 μM) without cytotoxic effect (CC_50_ > 5 μM).

## 3. Experimental

### 3.1. Chemicals and Reagents

The γ-^32^P-ATP isotope was purchased from Muromachi Yakuhin (Tokyo, Japan). Oligonucleotides were synthesized by Gene Design Inc. (Osaka, Japan). Bacterial alkaline phosphatase (BAPC75) was purchased from Takara Bio (Otsu, Japan). 6-hydroxy-2,2',4,4'-tetrabromodiphenyl ether (PBDE, **1**) was isolated from a marine sponge, and compound **2** was obtained by methylation of compound 1 with trimethylsilyldiazomethane. Bromophene **3**, triclosan (**4**), 2,4,6-tribromophenol (**5**), 3,3',5,5'-tetrabromobisphenol A (**6**), *O*-hydroxybiphenyl (**7**), 1,6-dibromo-2-naphthol (**10**), and 4,4'-isopropylidenebis[2-(2,6-dibromophenoxy)ethanol] (**14**) were purchased from Wako Pure Chemical (Osaka, Japan). poly(U) RNA, 2,3,5,6-tetrafluoro-4-(pentafluorophenyl)phenol (**11**), (*R*)-(+)-3-3'-dibromo-1,1'-bi-2-naphthol (**12**), (*R*)-(+)-3,3'-dibromo-5,5',6,6',7,7',8,8'-octahydro-1,1'-bi-2,2'-naphthalenediol (**13**), and 4-(4-bromophenyl)-2,6-diphenylphenol (**16**) were obtained from Sigma-Aldrich (St. Louis, MO, USA). (*R*)-(−)-6,6'-dibromo-1,1'-bi-2-naphthol (**8**) and tetrachlorobisphenol A (**9**) were purchased from TCI (Tokyo, Japan). 2-Hydroxy-2',3',4',5,5'-pentachlorobiphenyl (**15**) and 2,2',4,4'-tetrabromodiphenyl ether (**17**) were obtained from AccuStandard (New Haven, CT, USA).

### 3.2. Extraction of PBDE

The specimens used in this study were collected from marine organisms near Okinawa Islands, Japan (Table 1). Extractions were performed three times with either ethanol or acetone, and the ethyl-soluble portions (PM/SR-*-1) were obtained after concentration and partition. The aqueous layer was concentrated and methanol-soluble portions (PM/SR-*-2) were obtained by washing the residue and concentration.

### 3.3. Screening for HCV NS3 Helicase Inhibitors

The fluorescence helicase assay based on FRET was performed as described in our previous study [23]. The dsRNA substrate was prepared by annealing the 5' Alexa Fluor 488 labeled fluorescence strand (5'-UAGUACCGCCACCCUCAGAACCUUUUUUUUUUUUUU-3') to the 3' BHQ1 labeled quencher strand (5'-GGUUCUGAGGGUGGCCCUACUA-3') at a 1:2 molar ratio. The dsRNA substrate has the 3'-overhang that is necessary for NS3 helicase to bind RNA prior to the duplex unwinding. The capture strand (5'-TAGTACCGCCACCCTCAGAACC-3'), which is complementary to the quencher strand, prevents the unwound duplexes from reannealing. None of the above three strands is self-complementary. The fluorescence and quencher strands were purchased from Japan Bio Services (Saitama, Japan). The capture strand was purchased from Tsukuba Oligo Service (Ibaraki, Japan). The reaction mixture contained 25 mM MOPS-NaOH (pH 6.5), 3 mM MgCl_2_, 2 mM dithiothreitol, 4 U of RNasin (Promega, WI, USA), 50 nM dsRNA substrate, 100 nM capture strand, 5 mM ATP, an extract from a marine organism, and 240 nM NS3 in a total volume of 20 µL. Each extract from a marine organism diluted with DMSO was added to the reaction mixture at a final concentration in the range of 17.5–32.5 µg/mL. The full-length HCV NS3 protein with serine protease and ATPase/helicase was expressed and purified as described previously [23].

The reaction was started by adding HCV NS3 helicase and performed at 37 °C for 30 min using a LightCycler 1.5 (Roche Diagnostics, Basel, Switzerland). Fluorescence intensity was recorded every 5 s from 0 to 5 min, and then every 30 s from 5 to 30 min. Helicase activity was calculated as the initial reaction velocity relative to that of the control without a sample but with DMSO.

### 3.4. ATP Hydrolysis (ATPase) Assay

Following our previous report [26], unless otherwise stated, the standard assay reaction (10 μL) contained the following components: 25 mM MOPS-NaOH (pH 7.0), 1 mM DTT, 5 mM MgCl_2_, 5 mM CaCl_2_, 1 mM [γ-^32^P] ATP, 300 nM NS3, and 0.1 μg/μL poly(U) with serial dilution of the tested compounds in DMSO. Samples were incubated at 37 °C for 10 min, and the reaction was terminated by adding 15 μL of stop solution (10 mM EDTA). A small portion (2 μL) of reaction mixture was spotted on a PEI-cellulose TLC plate (Merck Millipore, Darmstadt, Germany) and developed by ascending chromatography in 0.75 M LiCl/1 M formic acid solution for 25 min. The TLC plate was then air-dried, and applied to autoradiography measured by an Image Reader FLA-9000 and quantified by Multi Gauge V3.11 software (Fujifilm, Tokyo, Japan). For the bacterial alkaline phosphatase assay, the buffer provided with the kit (Takara Bio, Otsu, Japan) was used, and then subjected to the assay as described above.

### 3.5. Gel Mobility Shift Assay (GMSA)

GMSA was performed with slight modification as described previously [26]. In brief, [γ-^32^P] ATP-labeled single-stranded RNA (0.4 nM) was incubated in a buffer containing 30 mM Tris-HCl pH 7.5, 100 mM NaCl, 2 mM MgCl_2_, 1 mM DTT, 20 U of RNasin plus (Promega) in the presence of 300 nM NS3 protein with serial dilution of PBDE in DMSO at room temperature for 15 min in a final reaction volume of 20 μL. The protein-RNA complexes were loaded onto a 6% native-PAGE (acrylamide:bis = 19:1) and after electrophoresis in TBE buffer, the labeled RNA bands were visualized and quantified with an Image Reader FLA-9000 (Fujifilm) and Multi Gauge V3.11 software (Fujifilm), respectively.

### 3.6. HCV Replication Assay

The cell lines harboring the subgenomic replicon RNAs of genotype 1b strain N [27] were seeded at 2 × 10^4^ cells per well in a 48-well plate 24 h before treatment. The cells were treated with PBDE at various concentrations for 72 h and lysed in cell culture lysis reagent (Promega). A luciferase assay system (Promega) was used to determine the luciferase activity, and the luminescence was measured using Luminescencer-JNR AB-2100 (ATTO, Tokyo, Japan), corresponding to the expression level of the HCV replicon.

### 3.7. Toxicity Assay

MTS assay was carried out to determine cytotoxicity using a CellTiter 96 aqueous one-solution cell proliferation assay kit (Promega) according to the manufacturer’s instructions.

## 4. Conclusions

In conclusion, the present study showed that PBDE (**1**) isolated from a marine sponge inhibited NS3 helicase through suppression of the ATPase and RNA binding activities. Moreover, PBDE (**1**) did not inhibit bacterial alkaline phosphatase, suggesting that PBDE (**1**) is specific for NS3 inhibition. Structure-activity relationships demonstrated that the biphenyl ring, bromine, and phenolic hydroxyl group on the benzene backbone might be crucial groups essential for the inhibitory potency. 
